# Acute exhaustive exercise regulates IL-2, IL-4 and MyoD in skeletal muscle but not adipose tissue in rats

**DOI:** 10.1186/1476-511X-10-97

**Published:** 2011-06-13

**Authors:** José C Rosa Neto, Fábio S Lira, Nelo E Zanchi, Lila M Oyama, Gustavo D Pimentel, Ronaldo VT Santos, Marília Seelaender, Cláudia M Oller do Nascimento

**Affiliations:** 1 Department of Physiology of Nutrition, Federal University of São Paulo, São Paulo, Brazil; 2 School of Physical Education and Sports, University of São Paulo, São Paulo, Brazil; 3 Department of Bioscience, Federal University of São Paulo, Baixada Santista Campus, São Paulo, Brazil; 4 Molecular Cell Biology Study Group, Department of Cell Biology and Development, Institute o Biomedical Sciences I, University of São Paulo, Brazil

## Abstract

**Background:**

The purpose of this study was to evaluate the effect of exhaustive exercise on proteins associated with muscle damage and regeneration, including IL-2, IL-4 and MyoD, in extensor digitorum longus (EDL) and soleus muscles and mesenteric (MEAT) and retroperitoneal adipose tissues (RPAT).

**Methods:**

Rats were killed by decapitation immediately (E0 group, n = 6), 2 (E2 group, n = 6) or 6 (E6 group, n = 6) hours after the exhaustion protocol, which consisted of running on a treadmill at approximately 70% of VO_2max _for fifty minutes and then at an elevated rate that increased at one m/min every minute, until exhaustion.

**Results:**

The control group (C group, n = 6) was not subjected to exercise. IL-2 protein expression increased at E0 in the soleus and EDL; at E2, this cytokine returned to control levels in both tissues. In the soleus, IL-2 protein expression was lower than that in the control at E6. IL-4 protein levels increased in EDL at E6, but the opposite result was observed in the soleus. MyoD expression increased at E6 in EDL.

**Conclusion:**

Exhaustive exercise was unable to modify IL-2 and IL-4 levels in MEAT and RPAT. The results show that exhaustive exercise has different effects depending on which muscle is analysed.

## Introduction

Exercise promotes physiological changes in response to disturbances in homeostasis [1]. These changes do not cease until a small overcompensation is attained, showing that exercise provides an excellent model for the study of physiological stress and the adaptive capacity of the body [2]. Acute and training exercises modulate cytokine levels in the serum [3] and other tissues, such as adipose tissue and skeletal muscle [4-6].

IL-2 and IL-4 are cytokines involved in immune response. IL-2, a pro-inflammatory cytokine, has multiple functions in the inflammatory response, including activation of immune cell effectors and stimulation of the number of white blood cells on the endothelial surface of skeletal muscle [7]. In addition, it is a potent inducer of the proliferation, differentiation, development, survival, memory and regulatory functions of T-lymphocytes [8].

In contrast, IL-4 is a pleiotropic cytokine that is important in the anti-inflammatory response. T-cells, mast cells and neutrophils are the main producers of IL-4 [9,10]. It has been reported that other cells, such as liver, fibroblasts, brain, and muscle cells, are able to express IL-4 [11,12].

Prokopchuk et al. [13] demonstrated that IL-4, IL-13, IL-4Rα and IL-13Rα1 are expressed in skeletal muscle and are up-regulated after strength training. IL-4 is involved in the regulation of muscle cell fusion and muscle growth through the IL-4 receptor.

Recently, we showed [4] that the IL-10/TNF-α ratio is increased in skeletal muscle, especially EDL, upon exercise but is decreased in adipose tissue. This result showed that exhaustive exercise has different effects; in muscle, it induces an anti-inflammatory effect, especially in type 2 fibres, while a pro-inflammatory effect occurs in adipose tissue.

Several studies have analysed the effect of chronic or acute exercise on IL-4 and IL-2 protein serum concentrations or on their production by immune cells. Some studies have found a decrease, and others have found no change, but most found increases, especially in serum concentrations of IL-2 [3,14-17].

IL-2 and IL-4 are important for the immune response because they result in repair of muscle damage elicited by exercise. However, the literature is inconsistent with respect to the effect of exercise on IL-4 and IL-2 production. In the present study, we examined the effects of acute exhaustive exercise on the time course of IL-2 and IL-4 protein expression in rodent soleus and extensor digitorum longus (EDL) muscles as well as in retroperitoneal and mesenteric white adipose tissue depots. The expression of MyoD, an important mediator of stem cell activation in skeletal muscle, was also evaluated in the EDL and soleus muscle.

## Methods

### Animals

The Experimental Research Committee of the São Paulo Federal University approved all procedures for the care of the animals used in this study. A total of 24 male Wistar rats 6 weeks in age (weighing ~250 g) were used. They were housed four per cage and received a chow diet and water *ad libitum *in an animal room under a 12 h light-dark cycle at 22 ± 1°C and 60 ± 5% humidity. The experiments were carried out after an acclimation period of one week.

### Experimental Design

The rats were killed by decapitation immediately (E0 group, n = 6), 2 (E2 group, n = 6) or 6 (E6 group, n = 6) hours after the exhaustion exercise protocol. The control group (C group, n = 6) was not subjected to the exercise protocol. Following sacrifice, the soleus and EDL muscles and mesenteric and retroperitoneal white adipose tissue were removed, snap frozen in liquid nitrogen, and stored at -80°C.

### Exercise protocol

All animals were accustomed to running on a rodent treadmill for 10 min per day for 4 days at a moderate level (5-10 m/min). On the fifth day, animals ran on the treadmill (20 m/min for fifty minutes and then at an elevated rate that increased at one m/min every minute) until exhaustion, defined as the moment where animals were unable to keep in pace with the treadmill. In the adaptation sessions, shock (9 mA) was utilised to promote learning. In the exhaustion test, however, the shock was not utilised.

### IL-2 and IL-4 protein determination

After euthanasia, the tissues (EDL, soleus, MEAT and RPAT) were rapidly removed and frozen. These tissues (0.1- 0.3 g) were homogenised in RIPA buffer (0.625% Nonidet P-40, 0.625% sodium deoxycholate, 6.25 mM sodium phosphate, and 1 mM ethylene-diamine tetraacetic acid at pH 7.4) containing 10 μg/ml of a protease inhibitor cocktail (Sigma-Aldrich, St. Louis, Missouri). Homogenates were centrifuged at 12.000 × *g *for 10 min at 4°C, the supernatant was saved, and the protein concentration was determined using a Bradford assay (Bio-Rad, Hercules, California) with bovine serum albumin as a reference. Quantitative assessment of IL-2 and IL-4 protein was carried out using ELISAs (DuoSet ELISA, BIOLEGEND). The sensitivity of the IL-2 and IL-4 test was found to be 5 pg/ml in the range of 40-1000 pg/ml. The intra- and inter-assay variabilities of the IL-2 and IL-4 kits were 2.2-6.1% and 5.4-8.8%, respectively.

### Protein analysis by western blotting

After euthanasia, the EDL and soleus were rapidly removed, homogenised in 1.0 ml extraction buffer (100 mM Trizma, 1% SDS, 100 mM sodium pyrophosphate, 100 mM sodium fluoride, 10 mM EDTA and 10 mM sodium orthovanadate) and boiled for 10 min. The extracts were then centrifuged at 12,000 rpm at 4°C for 40 min to remove the insoluble material. Determination of protein concentrations in the supernatants was performed using the Bradford dye method with a Bio-Rad reagent (Bio-Rad Laboratories, Hercules, CA, USA).

The proteins were treated with Laemmli sample buffer containing dithiothreitol and boiled for 5 min before loading onto 8% SDS-PAGE gels in a Bio-Rad miniature slab gel apparatus.

Aliquots containing similar amounts of protein (70 μg) were subjected to SDS-PAGE as described elsewhere (Carvalho et al. 1997). Electrotransfer of proteins from the gel to the nitrocellulose was performed for 1 h at 120 V (constant voltage) in a Bio-Rad miniature transfer apparatus. Nonspecific protein binding to the nitrocellulose was reduced by pre-incubation for 1 h at 22°C in blocking buffer (5% non-fat dry milk, 10 mM Tris, 150 mM NaCl and 0.02% Tween 20). The nitrocellulose membranes were incubated overnight at 4°C with antibodies against MyoD and α-Tubulin (obtained from Santa Cruz Biotechnology; Santa Cruz, CA, USA) diluted in blocking buffer with 1% bovine serum albumin (BSA) and then washed for 30 min in blocking buffer without BSA. The blots were subsequently incubated with a peroxidase-conjugated secondary antibody for 1 h at 22°C and processed for enhanced chemiluminescence to visualise the immunoreactive bands. Band intensities were quantified by optical densitometry (Scion Image-Release Beta 3b, NIH, USA) of the developed autoradiographs.

### Statistical analysis

Statistical analysis was performed using a commercially available statistical package from SigmaStat (version 3.1, SigmaStat, SYSTAT, Point Richmond, CA). The data are expressed as the means ± SE. Implementation of the Kolmogorov-Smirnov test revealed that the results of experiments were distributed normally. Intergroup comparisons were performed using a one-way ANOVA test. Post-hoc comparison tests between groups were done using the Holm-Sidak test. A p-value of less than 0.05 was considered statistically significant.

## Results

Mean time to exhaustion did not differ among groups.

### IL-2 and IL-4 protein levels in skeletal muscle

**Figure 1:**
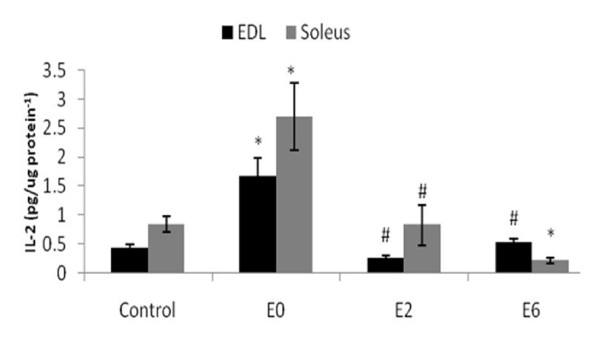
**IL-2 protein expression in the EDL and soleus muscle after exhaustive exercise at different times (0 [E0], 2 [E2] and 6 [E6] hours) after exhaustion and in the control group (C) by ELISA**. N = 6 for all groups. Values shown are the means ± SE. *, significantly different from all other groups (P < 0.05); #, significantly different from the E0 group (P < 0.05).

Figure 1 shows that IL-2 levels were increased in the EDL and soleus skeletal muscle at E0 and returned to control levels in the E2 and E6 soleus muscle. Additionally, six hours after exhaustion, the levels of IL-2 in the soleus muscle decreased relative to the levels in the other groups. The IL-2 protein content in the EDL and soleus muscles was similar.

**Figure 2:**
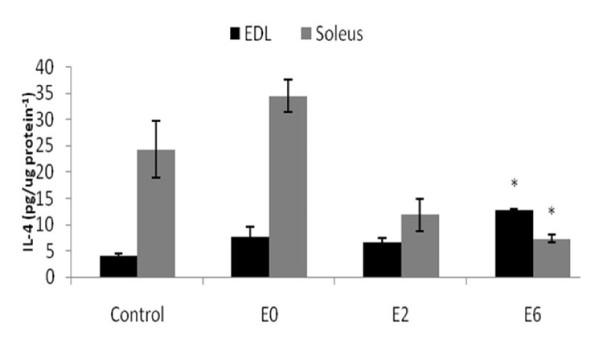
**IL-4 protein expression in the EDL and soleus muscle after exhaustive exercise at different times (0 [E0], 2 [E2] and 6 [E6] hours) after exhaustion and in the control group (C) by ELISA**. N = 6 for all groups. Values are shown the means ± SE. *, significantly different from all other groups (P < 0.05).

The IL-4 protein content was higher in the EDL at E6 compared with all other groups (Figure 2). The opposite effect was observed in the soleus muscle; IL-4 protein levels were lower at E6 compared with other groups (Figure 2). In the E0 group, the IL-4 protein content was higher in soleus than in EDL.

### MyoD protein levels in EDL and soleus muscle

**Figure 3:**
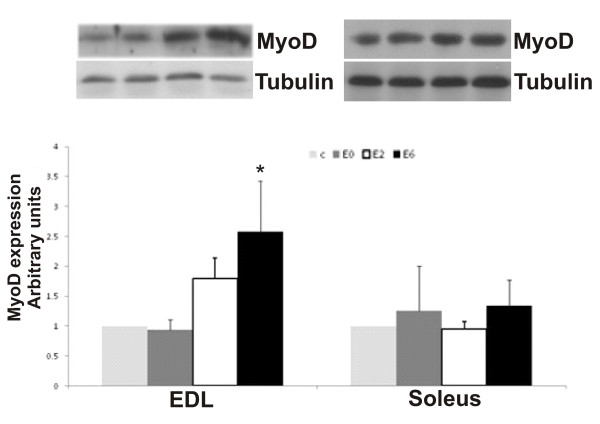
**Expression of MyoD in the EDL and soleus muscle after exhaustive exercise at different times (0 [E0], 2 [E2] and 6 [E6] hours) after exhaustion and in the control group (C)**. N = 6 for all groups. Values shown are the means ± SE. *, significantly different from all other groups (P < 0.05).

Exhaustive exercise increased MyoD expression 2.5-fold in the E6 group compared to the C and E0 groups (P < 0.001). At E2, the elevation was approximately 1.8-fold and was not statistically significant (P = 0.071). In the soleus muscle, exhaustive exercise did not change MyoD protein expression (Figure 3).

### IL-2 and IL-4 protein levels in adipose tissue

The exhaustive exercise protocol did not change the levels of IL-2 and IL-4 protein in RPAT and MEAT (Table 1). Compared to MEAT, RPAT had a higher IL-2 protein content at E0 and a higher IL-4 protein content at all time points (Table 1).

**Table 1:** Cytokine (IL-2 and IL-4) protein expression in the retroperitoneal (RPAT) and mesenteric (MEAT) adipose tissue after exhaustive exercise at different times (0 [E0], 2 [E2] and 6 [E6] hours) after exhaustion and in the control group (C) by ELISA

	C	E0	E2	E6
RPAT				

**IL-2**	4.25 ± 1.08	3.92 ± 0.38	4.13 ± 1.03	4.19 ± 1.2
**IL-4**	4.69 ± 0.43	5.62 ± 0.16	4.85 ± 0.38	3.93 ± 0.15

MEAT				

**IL-2**	1.99 ± 0.30	2.18 ± 0.36	2.12 ± 0.30	1.55 ± 0.20
**IL-4**	2.40 ± 0.21	1.91 ± 0.06	2.69 ± 0.18	1.86 ± 0.11

N = 6 for all groups. Values shown are the means ± SE.

## Discussion

In the present study, we demonstrate that exhaustive exercise promoted an increase in IL-2 protein concentration in EDL and soleus muscles, without changes in IL-4, at E0. However, six hours after exhaustion, the IL-4 protein content increased in the EDL and decreased in the soleus muscle. The increase in IL-4 protein in the EDL at E0 occurred at the same times as the increase in MyoD expression. In contrast, the adipose tissues depots analysed in this study showed no changes in these cytokines upon exhaustive exercise. We also demonstrated heterogeneity in protein content; for example, we observed higher IL-4 levels in the soleus compared to the EDL and in RPAT as compared to MEAT. This heterogeneity of cytokine levels in different adipose tissues and muscle depots has been demonstrated by others [4-6], although the present study demonstrates this under conditions of exhaustive exercise.

IL-4 expression was increased in the EDL and decreased in the soleus muscle six hours after exhaustion, suggesting that exercise activated different transcription factors depending on the type of muscle fibre [18,19].

The finding of increased IL-4 levels in type II muscle fibres (EDL) is in agreement with other studies showing an increase in IL-4 mRNA and IL-4 receptor mRNA and protein in human *triceps brachii *skeletal muscle (a type II fibre muscle) after training [13].

Recent reports indicate that IL-4 promotes myoblast recruitment, fusion and growth and that this cytokine acts as a pro-migratory agent for myogenic cells [20]. In this sense, both *in vivo *and *in vitro*, a lack of IL-4 or IL-4 receptors causes a reduction in muscle size and in the quantity of myonuclei [8]. The regulation of myoblast fusion and myotube maturation is important for the maintenance and repair of adult muscle.

It is well known that muscle fibres damaged by exhaustive exercise trigger pro- and anti-inflammatory cytokine release [4,21,22]. Thus, the increase in IL-4 protein content in EDL six hours after exhaustive exercise may be important for myotube growth and repair.

To confirm this hypothesis, we evaluated the expression of MyoD, which is important for skeletal muscle stem cell activation and proliferation. Six hours after exhaustive exercise, the level of this protein was increased. This finding shows that exhaustive exercise is able to increase the proliferation of stem cells in skeletal muscle, which could contribute to muscle hypertrophy.

IL-2 protein expression was higher in the EDL than in the soleus muscle during the recovery from exhaustive exercise. As shown by Edwards et al. [7], IL-2 activates immune cell effectors and increases the white blood cells on the endothelial surface of skeletal muscle. This increase in IL-2 protein expression after exhaustive exercise, which we observed mainly in EDL, could elicit an immune response to injury in this tissue. Importantly, it is unknown whether IL-2 is produced by the skeletal muscle or by the immune cells infiltrating the muscle during exhaustive exercise. In studies of animal muscles after acute exercise, 85% showed intramuscular neutrophil infiltration [23].

In a previous study, we showed that exhaustive exercise caused an increase in IL-6 and TNF-α, which are pro-inflammatory cytokines that stimulate lipolysis to provide energy for contracting muscle [4]. To our knowledge, IL-2 and IL-4 do not have any metabolic effects. The fact that their concentrations were not modified by exhaustive exercise corroborated our previous idea that the cytokine alterations in adipose tissue during exercise occur to supply energetic fuel to the muscle.

In conclusion, acute exhaustive exercise modulates IL-2 and IL-4 expression in skeletal muscle. This regulation depends on the skeletal muscle fibre type. The increases in IL-4 and MyoD protein expression observed in the EDL may be related to increased activation and proliferation in stem cells after exhaustive exercise, which could contribute to muscle hypertrophy. The protein expression of various cytokines (IL-2, IL-4) in adipose tissue depots (MEAT and RPAT) is not regulated by exercise.

## Conflicts of interests

The authors declare that they have no competing interests.

## Authors' contributions

JCRN, FSL, NEZ, LMO, GDP, RVTS, MS, and CMON participed the sample collected, assess samples, design of the study and performed the statistical analysis, and writing of paper. All authors read and approved the final manuscript.
